# Flightless I Alters the Inflammatory Response and Autoantibody Profile in an OVA-Induced Atopic Dermatitis Skin-Like Disease

**DOI:** 10.3389/fimmu.2018.01833

**Published:** 2018-08-10

**Authors:** Zlatko Kopecki, Natalie E. Stevens, Heng T. Chong, Gink N. Yang, Allison J. Cowin

**Affiliations:** Regenerative Medicine, Future Industries Institute, University of South Australia, Adelaide, SA, Australia

**Keywords:** atopic dermatitis, flightless I, autoantibody, inflammation, skin barrier

## Abstract

Atopic dermatitis (AD) is a chronic pruritic inflammatory skin disease characterized by excessive inflammation and disrupted skin barrier function. Although the etiology of AD is not completely understood, clinical and basic studies suggest increasing involvement of autoantibodies against intracellular proteins. An actin remodeling protein, Flightless I (Flii), has been shown to promote development of inflammatory mediated skin conditions and impairment of skin barrier development and function. Here, we sought to determine the effect of altering *Flii* expression on the development of AD and its contribution to autoimmune aspects of inflammatory skin conditions. Ovalbumin (OVA)-induced AD skin-like disease was induced in *Flii* heterozygous (*Flii^+/−^*), wild-type (*Flii^+/+^*), and *Flii* transgenic (*Flii^Tg/Tg^*) mice by epicutaneous exposure to OVA for 3 weeks; each week was separated by 2-week resting period. Reduced *Flii* expression resulted in decreased disease severity and tissue inflammation as determined by histology, lymphocytic, and mast cell infiltrate and increased anti-inflammatory IL-10 cytokine levels and a marked IFN-γ Th_1_ response. In contrast, *Flii* over-expression lead to a Th_2_ skewed response characterized by increased pro-inflammatory TNF-α cytokine production, Th_2_ chemokine levels, and Th_2_ cell numbers. Sera from OVA-induced AD skin-like disease *Flii^+/−^* mice showed a decreased level of autoreactivity while sera from *Flii*^Tg/Tg^ mice counterparts showed an altered autoantibody profile with strong nuclear localization favoring development of a more severe disease. These findings demonstrate autoimmune responses in this model of OVA-induced AD-like skin disease and suggest that Flii is a novel target, whose manipulation could be a potential approach for the treatment of patients with AD.

## Introduction

Atopic dermatitis (AD) is one of the most common heterogeneous inflammatory skin diseases affecting 20% of children and 1–3% of adults worldwide (1). The disease is associated with impairments in the skin barrier and variable clinical indicators including occurrence of eczematous lesions, pruritus, and cheilitis (1). The etiology of AD is complex and is often characterized by abnormal immunological pathways that manifest in an imbalance of T-helper (Th)_1_ and Th_2_ responses (2). Typically, AD is described as having a biphasic course consisting of an acute inflammatory Th_2_-dominated phase associated with IgE production and a chronic phase distinguished by reappearance of Th_1_ responses, tissue remodeling, and dermal thickening (3). Histopathologically, AD is characterized by an inflammatory infiltrate consisting of CD4^+^ memory T cells, mast cells, and eosinophils and a controlled temporal-spatial expression of pro-inflammatory cytokines and chemokines driving atopic inflammation of the skin (4).

Research within the last decade has found an association between AD and autoantibody development, suggesting the contribution of autoantibodies to the pathogenesis of AD (3, 5–8). It is proposed that tissue damage induced by AD allows exposure of intracellular antigens that are normally inaccessible by antibodies to the extracellular space, where they can interact with B cells and antibodies (9). A broad spectrum of IgE targeting self-antigens have been identified in above 90% of severe AD patients and high-avidity IgG autoantibodies have been proposed as potential diagnostic markers for severe AD (5, 10–12). The fact that these autoantibodies are associated with disease severity implicates their role in both humoral and cellular immunity in AD pathogenesis (5, 12). Antinuclear antibodies have been shown to have both biological and clinical significance acting as sensors of cellular stress and inflammation associated with environmental factors (13). While the presence of autoantibodies can have a protective role as natural autoantibodies (14), the presence of nuclear autoantibodies in AD has been suggested to lead to the continual provocation of the immune system hence contributing to the severity and chronicity of disease.

Flightless I (Flii) is a highly conserved and unique member of the gelsolin family of actin remodeling proteins and a nuclear receptor activator affecting the transcriptional activity of many modulators of tissue remodeling and inflammation (15, 16). Flii has been demonstrated to regulate cytokine secretion and cellular inflammatory responses *via* its intracellular and extracellular effects on toll-like receptors (17–20). Flii expression increases in skin during development and in response to inflammation, injury, skin cancer development, wound healing, and skin blistering (21–23). Over-expression of *Flii* delays the development of an intact skin barrier in the embryonic skin of mice and impairs the recovery of the epidermal barrier post injury *via* its effects on tight-junction formation (22). A recent study has shown that reducing Flii levels either genetically or using Flii neutralizing antibodies decreases erythema, inflammatory cell infiltrate, and pro-inflammatory cytokine secretion in a mouse model of psoriasiform dermatitis (24). Taken together, these findings suggest a possible role for Flii in inflammatory responses mediating AD. Using an OVA-induced AD-like skin mouse model (25), this study aimed to investigate the effect of differential *Flii* gene expression on development of AD *via* characterization of inflammatory and autoimmune responses responsible for AD pathogenesis.

## Materials and Methods

### Animal Studies

Mice were maintained according to the Australian Code for the Care and Use of Animals for Scientific Purposes under protocols approved by the Child Youth and Women’s Health Service Animal Ethics Committee (AEC916/06/2015). Mice with the BALB/c background and wild-type controls were obtained from inbred litters. *Flii*-deficient heterozygous null mice (*Flii^+/−^*) and mice carrying the complete human *Flii* gene on a cosmid transgene were maintained as described previously (26, 27). Heterozygous transgenic mice *Flii^Tg/+^* were made by crossing *Flii^+/+^* with cosmid transgene *Flii^+/−^*. These transgenic mice were intercrossed to obtain animals homozygous for the transgene *Flii^Tg/Tg^* which carry two copies of the mouse *Flii* gene and two copies of the human *Flii* transgene.

**Figure 1:**
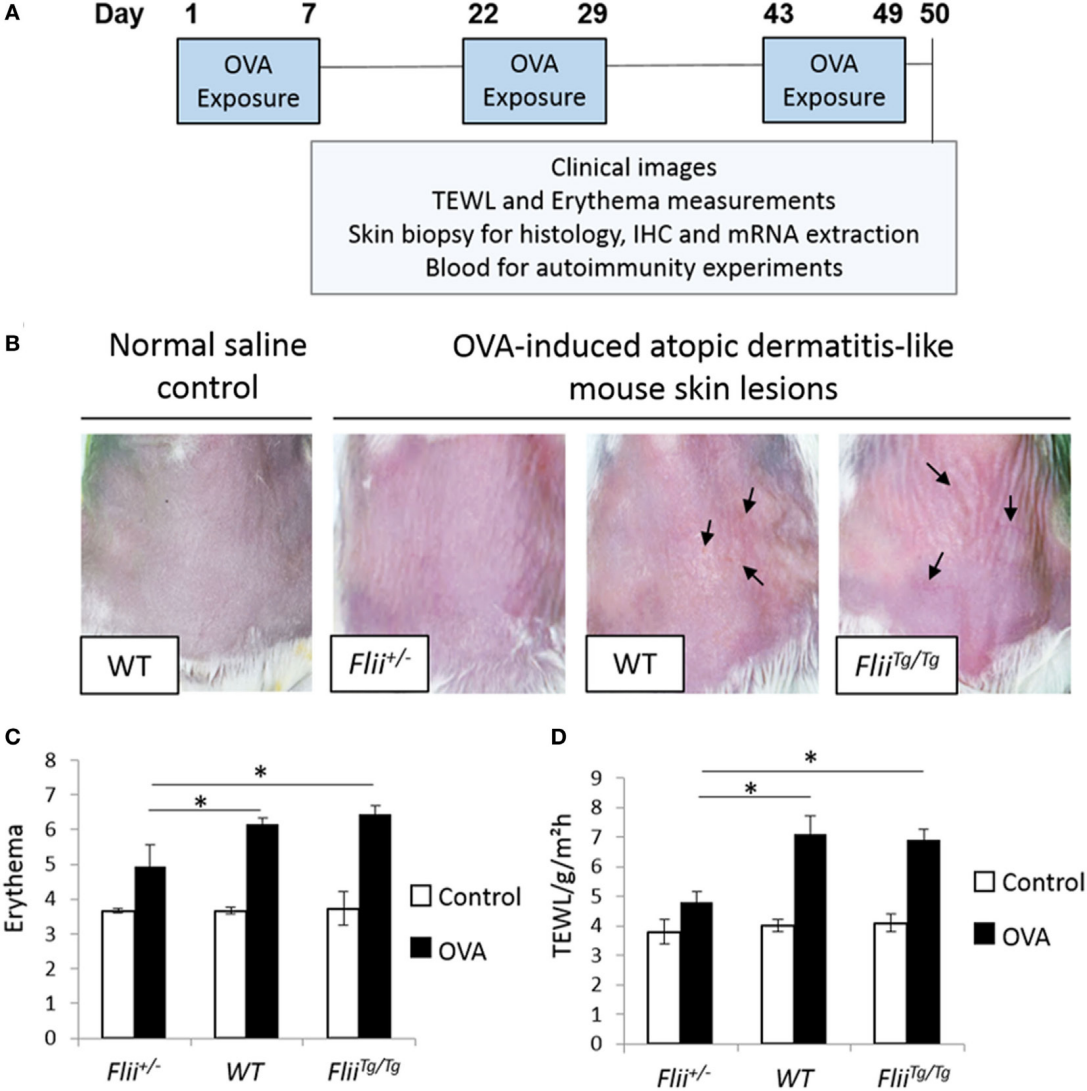
Reduced *Flii* expression leads to decreased development of OVA-induced atopic dermatitis (AD)-like lesions. **(A)** Ovalbumin (OVA) exposure protocol, including a total of three 1-week exposures to 100 µl 0.1% OVA or saline control on patches separated from each other by 2-week resting interval. **(B)** Representative clinical images of OVA-induced AD-like skin lesions in mice differential *Flii* expression illustrating high degree of erythema, skin thickening, and scaling (black arrowhead) in WT and *Flii^Tg/Tg^* mice exposed to OVA. **(C,D)** OVA exposure leads to development of AD-like lesions demonstrated by increased erythema and transepidermal water loss (TEWL). Decreasing *Flii* expression reduces development of AD-like lesions with significantly decreased erythema and TEWL measurements compared to OVA exposed skin of WT and *Flii^Tg/Tg^* animals. *n* = 8. Mean ± SEM. *<0.05.

Atopic dermatitis was induced *via* epicutaneous exposure of mice to OVA as previously described (25). Briefly, 10- to 12-week-old female wild-type (*Flii^+/+^*), *Flii* heterozygous (*Flii^+/−^*), and *Flii* transgenic (*Flii^Tg/Tg^*) mice (*n* = 8/genotype) were anesthetized using isoflurane inhalation, the skin on the back of mice was shaved and then tape stripped four times by a transparent adhesive tape (Tegaderm) to introduce a standardized skin injury. A gauze patch (1 × 1 cm^2^) soaked with 100 µl of 0.1% OVA (OVA group) in saline or 0.9% saline (control group) was placed on the back skin and secured with Tegaderm dressing. The experiment comprised three 1-week exposures with a 2-week interval between each exposure week (Figure 1A). Clinical images of affected skin, transepidermal water loss (TEWL), and erythema measurements were recorded at the end of the third sensitization week. The level of skin erythema was measured daily using a handheld DermaLab Unit (Cortex Technology) following manufacturer’s instructions. This instrument uses skin reflectance spectroscopy to determine the redness of inflamed skin. The instrument was blanked prior to placing a probe directly onto the inflamed AD-like lesions on dorsal skin and a reading obtained as previously described (24). Measurements of TEWL were obtained with a calibrated Vapometer evaporimeter (Delfin Technologies, Finland). The probe was allowed to equilibrate for approximately 30 s before a brief measurement period (8 s) as indicated in manufacturer’s guidelines. The Vapometer was placed directly onto the inflamed AD-like lesions on dorsal skin (using an 11-mm adaptor) and held there securely for the duration of the measurement. For both erythema and TEWL analysis, three separate measurements were taken per mouse to ensure the entire region of AD-like inflamed back skin was assessed as previously described (24). Mice were then euthanized and skin biopsies collected for histology analysis, immunohistochemistry, and mRNA extraction. Blood was collected for autoimmunity experiments.

### Histology and Immunohistochemistry

Paraffin-embedded fixed tissue samples were stained with hematoxylin and eosin or toluidine blue following established protocols (28). Level of skin inflammation was assessed using skin thickness measurements, and analysis of inflammatory and mast cell numbers in lesional skin of OVA-induced AD-like skin lesions of *Flii^+/−^*, wild-type, and *Flii^Tg/Tg^* mice using high magnification images and light microscopy using optimized protocols using Image Pro-Plus 5.1 program (MediaCybernetics Inc.) as previously described (24). Skin thickness included epidermal and dermal measurements of OVA-induced AD-like lesions; inflammatory cell number was assessed by counting total inflammatory cells in 10 different HPF of view in OVA-induced AD-like lesions, and mast cell analysis included counting toludine blue positive mast cells in entire sections of OVA-induced AD-like skin lesions per mm^2^. Immunohistochemistry was also performed on paraffin-embedded fixed AD-like skin lesions following antigen retrieval according to the manufacturer’s protocols (DAKO Corporation, Glostrup, Denmark). Following blocking in 3% normal goat serum, primary antibodies against CD4 [rat monoclonal, #14-9766-82 (Thermo Scientific, Australia) (1:200)], T-bet [rabbit polyclonal, #PA5-40573 (Thermo Scientific, Australia) (1:100)], GATA-3 [rabbit polyclonal, #ab106625 (Abcam Australia) (1:100)], and ROR-γ [rabbit monoclonal, #ab207082 (Abcam Australia) (1:3,000)] were applied and slides were incubated at 4°C overnight in a humidified chamber before application of species-specific, Alexa Flour-488 or Alexa Flour-594 secondary antibodies (Invitrogen, Australia) for 1 h at room temperature. Finally, slides were washed and mounted in Fluorescence Mounting Medium (Dako, Australia). Images were captured on Olympus microscope and CellSense Live Science Imaging Software program (Olympus, Germany) used for counting the positive cells in the AD-like lesions of *Flii^+/−^*, wild-type, and *Flii^Tg/Tg^* mice. Negative controls included replacing primary antibodies with normal rabbit IgG, or normal mouse IgG. For verification of staining, non-specific binding was determined by omitting primary or secondary antibodies. All control sections had negligible immunofluorescence.

### RTq-PCR

Harvested tissue was snap-frozen in liquid nitrogen and total RNA was isolated using Ultraclean Tissue and Cell RNA Isolation Kit (MoBio Laboratories, Carlsbad, CA, USA) according to the manufacturer’s protocol. Total cDNA was synthesized using iScript cDNA Synthesis Kit (Bio-Rad Laboratories, Hercules, CA, USA) according to manufacturer’s protocol. Quantitative PCR was performed using iQ SYBR Green Supermix (Bio-Rad Laboratories, Hercules, CA, USA) in triplicate reactions. The plates were placed in a CFX Connect Real-Time PCR Detection System (Bio-Rad Laboratories, Hercules, CA, USA). Reactions underwent 30 s at 95°C, then 40 cycles of 5 s at 95°C, 20 s at 60°C, and 10 s at 95°C before determination of melt curve between 65 and 95°C. GAPDH and CyPA were used as reference genes and inter-reaction calculator method was applied for all plates. For relative comparison, the cycle threshold value (Ct) was analyzed using the ΔΔCt method and data reported as Ct normalized to reference genes. Sequences for PCR primers are listed in Table S1 in Supplementary Material.

### Autoantibody Immunofluorescence

In order to assess the degree of autoimmunity in OVA-induced AD-like skin of *Flii^+/−^*, wild-type, and *Flii^Tg/Tg^* mice, sub-confluent primary wild-type mouse keratinocytes were stained with mouse sera of AD-induced mice following established protocols (13, 29). Briefly, primary keratinocytes were isolated from murine epidermis as previously described (30), grown on glass coverslips and washed in 1× phosphate-buffered saline before paraformaldehyde (4%) fixation (10 min at room temperature). Fixed cells were subsequently permeabilized using 0.2% Triton-X-100 and 0.5% BSA in 1× phosphate-buffered saline (5 min at room temperature) before incubation with pooled murine serum (*n* = 3) diluted (1 in 10) in 0.5% BSA in 1× phosphate-buffered saline for 1 h. Bound murine IgG was detected with Alexa Flour 633 goat anti-mouse IgG (1 in 1,000; #A21050; Invitrogen, Mulgrave, VIC, Australia) in 1× phosphate-buffered saline for 1 h at room temperature. Following repeated 2 min washes with 1× phosphate-buffered saline, cells were stained with DAPI nucleic acid stain (Sigma-Aldrich) for 5 min at room temperature before washing and mounting for imaging. Negative controls included omitting the incubation with mouse serum. All control sections had negligible immunofluorescence. Staining pattern of autoantibodies was assessed using the Olympus microscope and CellSense Live Science Imaging Software program (Olympus, Germany).

### Immunoblot Analysis of AD Serum Autoreactive Antibodies

Protein was extracted from *WT* murine keratinocytes using standard protein extraction protocols (31). Samples of extracted protein (20 µg) were run on 10% SDS-PAGE gels (30 min; 200V) and transferred to nitrocellulose by wet transfer (1 h; 100V). Membranes were cut into strips, blocked in 3% bovine serum albumin (Sigma) for 30 min, and probed with murine serum diluted (1 in 10) in tris-buffered saline containing 3% BSA and 0.1% Tween overnight. After washing, horseradish peroxidase-conjugated goat anti-murine immunoglobulin secondary antibody was added for a further 1 h at room temperature. Stringent washes were performed before detection of horseradish peroxidase and exposure using GeneSnap analysis program (SynGene, Frederick, MD, USA).

### Statistical Analysis

Statistical differences were determined using the Student’s *t*-test or one-way ANOVA. For data not following a normal distribution, the Mann–Whitney *U* test was performed. A *P* value of less than 0.05 was considered significant.

## Results

### Flii-Deficient Mice Exhibit Reduced OVA-Induced AD-Like Skin Disease

The repeated epicutaneous exposure of OVA (Figure 1A) on mouse skin results in an AD-like skin disease with histological and phenotypic features of human AD, including erythema, skin thickening, and a localized immune response (25). *Flii* homozygous (*Flii^−/−^)* mice are embryonic lethal (26); therefore, using the OVA-induced AD-like skin mouse model, the severity of OVA-induced AD-like lesions was determined in response to different *Flii* gene levels in *Flii* heterozygous (*Flii^+/−^*), normal (*Flii^+/+^*), and *Flii* transgenic (*Flii^Tg/Tg^*) mice. All three mice genotypes developed localized inflammation which increased following each week of OVA exposure. *Flii*-deficient mice showed reduced levels of inflammation and scaling (Figure 1B). Spectrophotometric measurement of the redness of the OVA exposed skin showed that *Flii*-deficient mice had significantly less erythema (Figure 1B) than wild-type and *Flii^Tg/Tg^* counterparts at day 50 of the experiment. Similarly, the degree of TEWL was also significantly reduced in *Flii^+/−^* mice and very similar to that observed in control animals (Figure 1C). Control mice administered saline only showed no evidence of AD-like inflammation macroscopically or any development of erythema or TEWL (Figures 1B–D).

**Figure 2:**
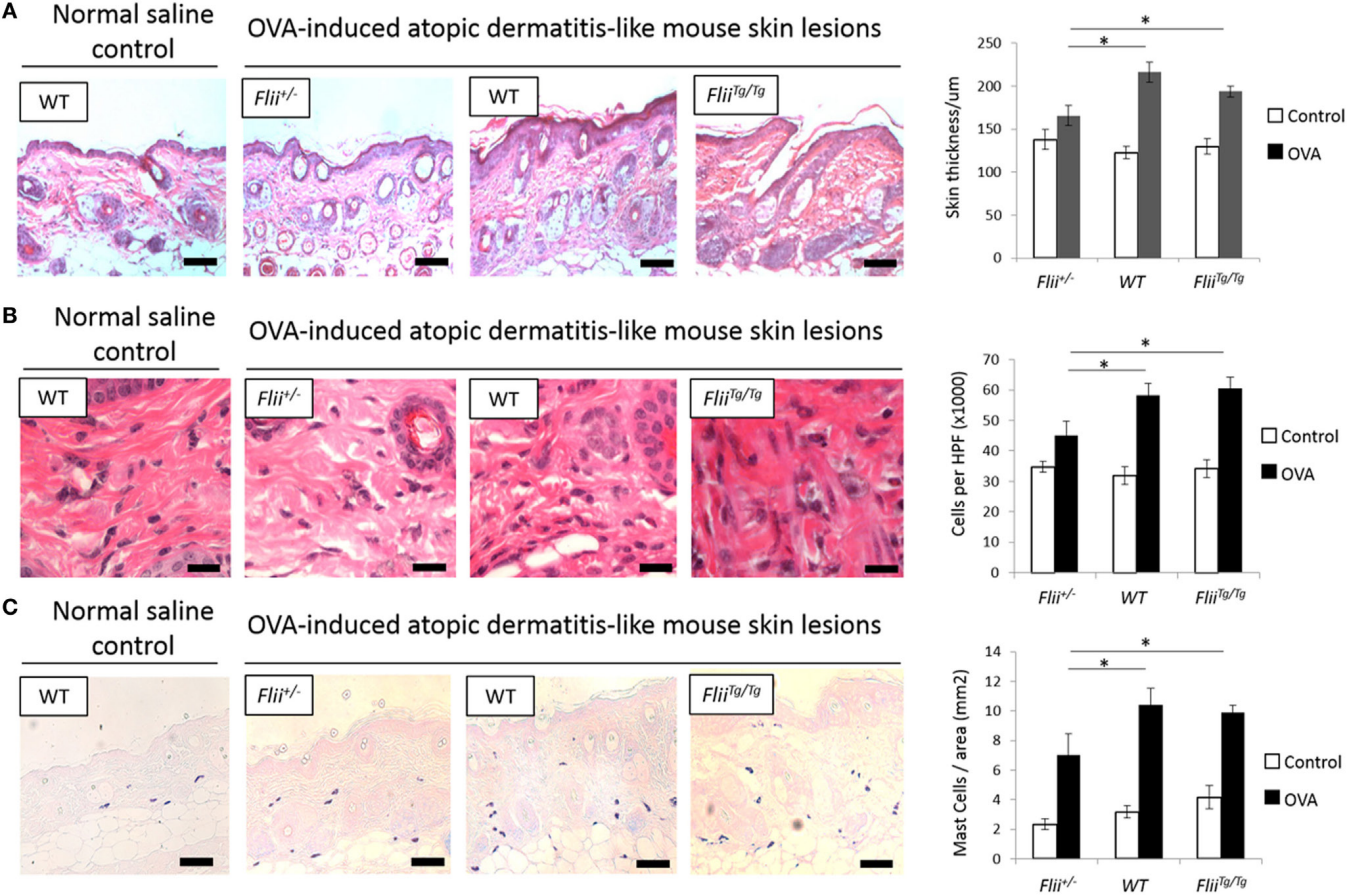
Mice with reduced *Flii* expression develop less severe OVA-induced atopic dermatitis (AD) skin-like lesions and tissue inflammation. **(A)** Representative images and graphical analysis of skin thickness in *Flii^+/−^*, WT, and *Flii^Tg/Tg^* mice with OVA-induced AD skin-like lesions illustrating evidence of epidermal hyperplasia, edema, and lymphocytic infiltrate. Magnification 4× scale bar 200 µm. **(B)** Representative images and graphical analysis of lymphocytic inflammatory infiltrate in in *Flii^+/−^*, WT, and *Flii^Tg/Tg^* mice with OVA-induced AD skin-like lesions illustrating decreased inflammation in *Flii^+/−^* mice skin. Magnification 1,000× scale bar 25 µm. **(C)** Representative images and graphical analysis of mast cells numbers in *Flii^+/−^*, WT, and *Flii^Tg/Tg^* mice with OVA-induced AD skin-like lesions illustrating decreased inflammation in *Flii^+/−^* mice skin. Magnification 4× scale bar 200 µm. *n* = 8. Mean ± SEM. *<0.05.

A hallmark of AD is skin thickening with a marked influx of leukocyte and mast cell inflammatory infiltrate. All OVA-induced AD-like skin sections showed evidence of AD compared to control saline exposed skin including a degree of epidermal hyperplasia and edema coupled with inflammatory lymphocytic and mast cell dermal infiltrate (Figures 2A–C). Examining the skin thickness in OVA-induced AD-like skin lesions revealed that deficient *Flii* mice had significantly thinner skin than OVA-induced AD-like skin lesions of wild-type and *Flii^Tg/Tg^* mice (Figure 2A). Assessment of lymphocytic and mast cell dermal infiltrate showed that *Flii* deficiency consistently resulted in significantly decreased inflammatory cell numbers (Figures 2B,C). In contrast, both normal and increased *Flii* gene expression resulted in thickened epidermis, increased numbers of inflammatory cells (Figure 2B) and mast cells (Figure 2C). Control mice skin exposed to saline only gauze patch showed no evidence of AD-like dermatitis features microscopically and had low levels of inflammatory infiltrate in the dermis (Figures 2A–C).

### Anti-Inflammatory Cytokine mRNA Profiles Are Increased in OVA-Induced AD-Like Skin of *Flii*-Deficient Mice

**Figure 3:**
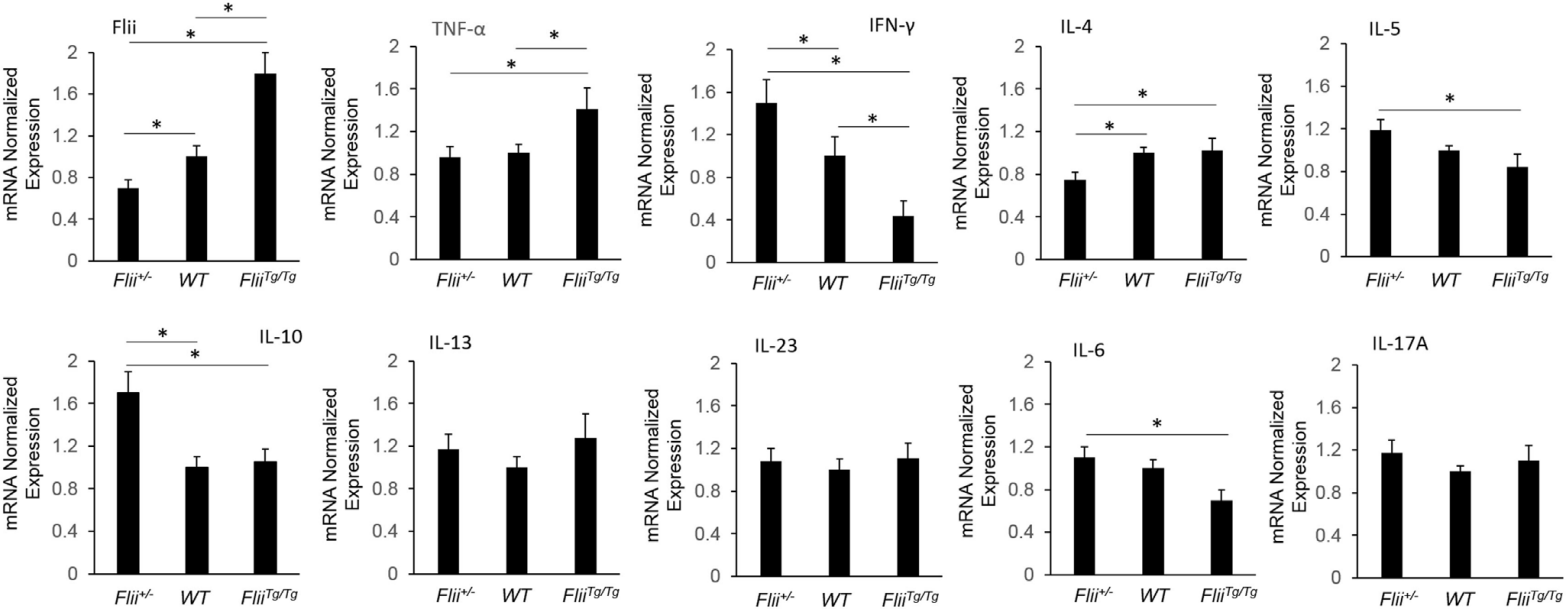
OVA-induced atopic dermatitis (AD) skin-like lesions of *Flii*-deficient mice exhibit increased anti-inflammatory cytokine levels. mRNA levels of Flii, pro-inflammatory, and anti-inflammatory cytokines responsible for manifestation of AD were analyzed in the OVA-induced AD skin-like lesions of *Flii^+/−^*, wild-type, and *Flii^Tg/Tg^* animals. OVA-induced AD skin-like lesions of *Flii*-deficient mice have significantly higher IFN-γ, reduced IL-4 signaling, and significantly higher levels of anti-inflammatory IL-10 while the *Flii* transgenic counterparts show significantly increased TNF-α and significantly reduced IFN-γ compared to wild-type animals. *n* = 6. Mean ± SEM. *<0.05.

**Figure 4:**
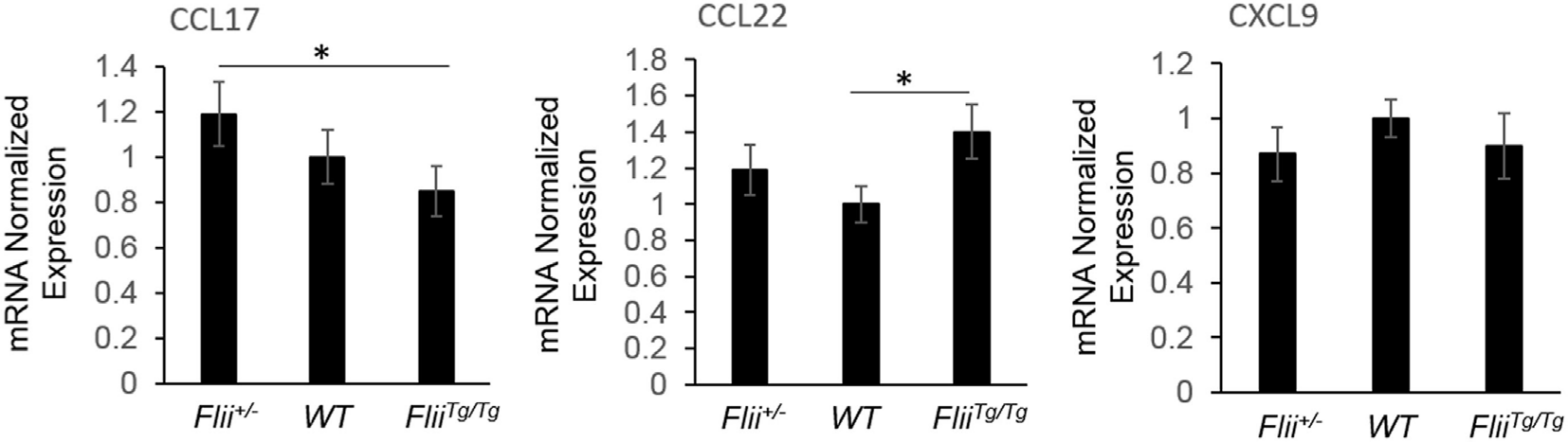
*Flii* expression affects Th_2_ chemokine levels in OVA-induced atopic dermatitis (AD) skin-like lesions. mRNA levels of CCL17, CCL22, and CXCL9 chemokines responsible for manifestation of AD were analyzed in the OVA-induced AD skin-like lesions of *Flii^+/−^*, wild-type, and *Flii^Tg/Tg^* animals. OVA-induced AD skin-like lesions of *Flii^+/−^* mice have significantly higher levels of CCL17 compared to *Flii^Tg/Tg^* counterparts, while CCL22 was significantly increased in OVA-induced AD skin-like lesions of *Flii^Tg/Tg^* mice compared to wild-type controls. *n* = 6. Mean ± SEM. *<0.05.

**Figure 5:**
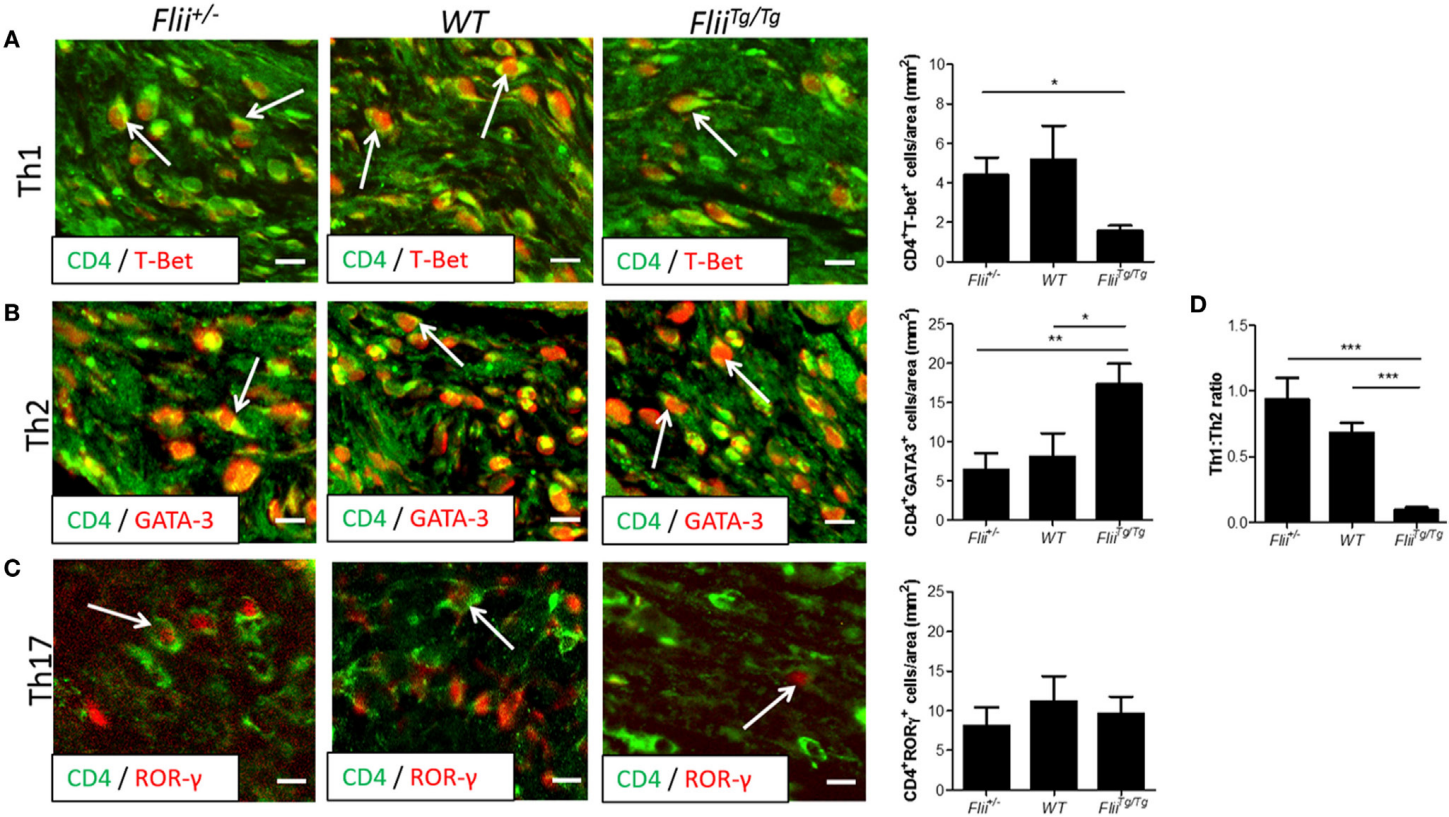
Mice with increased Flii expression exhibit increased Th2 cell numbers within OVA-induced atopic dermatitis (AD) skin-like lesions. **(A)** Representative images and graphical analysis of Th1 (CD4^+^T-bet^+^) cell numbers in AD skin-like lesions within *Flii^+/−^*, WT, and *Flii^Tg/Tg^* mice. **(B)** Representative images and graphical analysis of Th2 (CD4^+^GATA-3^+^) cell numbers in AD skin-like lesions within *Flii^+/−^*, WT, and *Flii^Tg/Tg^* mice. **(C)** Representative images and graphical analysis of Th17 (CD4^+^RORγ^+^) cell numbers in AD skin-like lesions within *Flii^+/−^*, WT, and *Flii^Tg/Tg^* mice. **(D)** Ratio of Th1 to Th2 cells within AD skin-like lesions of *Flii^+/−^*, WT, and *Flii^Tg/Tg^* mice. Magnification 4× scale bar 200 µm. *n* = 6. Mean ± SEM. *<0.05.

OVA-induced AD-like skin lesions of *Flii^+/−^*, wild-type, and *Flii^Tg/Tg^* mice were assessed for *Flii* and levels of cytokines and chemokines mediating the Th_1_ and Th_2_ inflammatory responses during AD pathogenesis. *Flii-*deficient mice showed approximately 25% decrease in *Flii* levels, while *Flii* over-expressing mice had a twofold increase in *Flii* levels compared to wild-type counterparts (Figure 3). *Flii* gene levels were found to affect number of key cytokines and chemokines responsible for development of AD-like lesions. Notably, main *Flii* deficiency favored a Th_1_ immune response and decreased inflammation with significant threefold increase in IFN-γ mRNA levels compared to *Flii^Tg/Tg^* mice, decreased pro-inflammatory IL-4, and increased anti-inflammatory IL-10 mRNA levels compared to both wild-type and *Flii^Tg/Tg^* mice (Figure 3). Interestingly, *Flii^+/−^* OVA-induced AD-like skin lesions also showed increased IL-5 and IL-6 mRNA levels when compared to *Flii^Tg/Tg^* mice but not wild-type counterparts (Figure 3). In contrast, over-expression of *Flii* resulted in similar cytokine levels to wild-type counterparts, except significantly increased TNF-α mRNA expression and significantly reduced IFN-γ mRNA expression when compared to both *Flii*-deficient and wild-type mice, which would favor more severe AD manifestation. Cytokine mRNA levels of IL-13, IL-23, and IL-17A did not differ between OVA-induced AD-like skin lesions of three genotypes (Figure 3). Expression of CCL22 mRNA was significantly increased in OVA-induced AD-like skin lesions of *Flii^Tg/Tg^* mice compared to wild-type mice, while OVA-induced AD-like skin lesions of *Flii^+/−^* mice showed elevated CCL17 chemokine mRNA expression compared to *Flii^Tg/Tg^* mice (Figure 4). CXCL9 chemokine mRNA levels were found to be similar in all three genotypes (Figure 4). The unbalanced skewed ratio of Th1/Th2 response in *Flii^Tg/Tg^* mice was confirmed by immunohistochemical co-staining of T cell subset markers, namely Th1 (CD4 and T-bet), Th2 (CD4 and GATA-3), and Th17 (CD4 and ROR-γ) showing increased Th2 cell numbers in dermal area of *Flii^Tg/Tg^* mice (Figures 5A–D).

### *Flii^Tg/Tg^* OVA-Induced AD-Like Disease Mice Have Altered Autoreactivity Profiles Compared With WT and *Flii^−/−^* Mice

**Figure 6:**
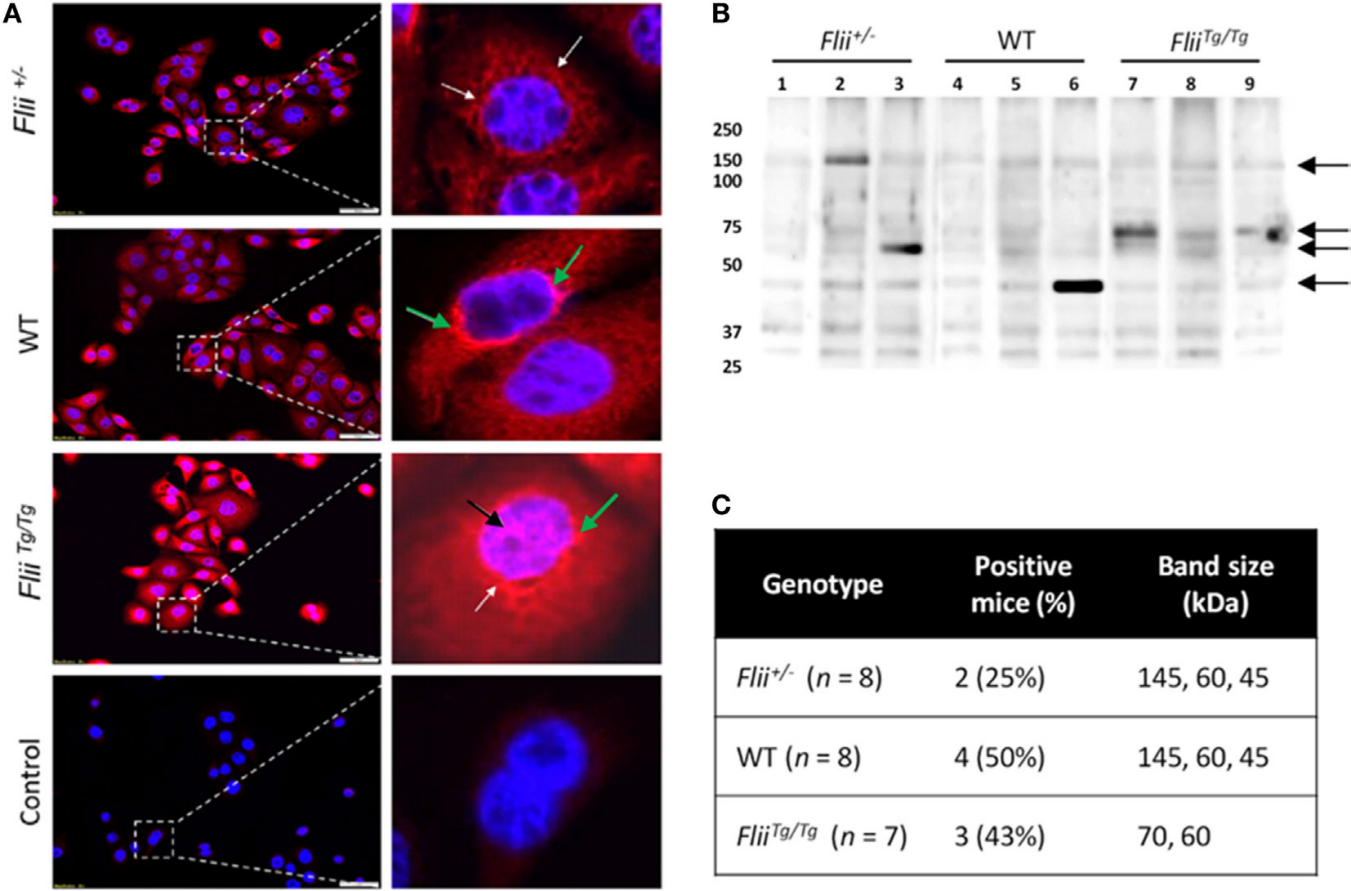
Over-expression of *Flii* produces an altered autoantibody profile in murine OVA-induced atopic dermatitis (AD) skin-like disease. **(A)** Representative images of IF microscopy staining patterns produced by mouse autoreactive IgG (red) and DAPI nuclear counterstaining (blue) in primary mouse keratinocytes. Magnification 20×; scale bar 50 µm. Antibodies from OVA-induced AD skin-like lesions of *Flii^+/−^* and WT mice producing a predominantly cytoplasmic (white arrow) and perinuclear (green arrow) staining patterns, while antibodies from OVA-induced AD skin-like lesions of *Flii^Tg/Tg^* mice produce a strong cytoplasmic, perinuclear (green arrow), and nuclear (black arrow) staining patterns. **(B)** Western blot analysis of murine keratinocyte proteins probed with pooled sera from OVA-induced AD-like mice (2–3 mice per lane); black arrows represent regions of autoreactivity. **(C)** Autoreactivity summary of Western blot results from murine keratinocyte lysate probed with sera from individual mice.

To compare autoantibody formation in the OVA-induced AD-like disease model between *Flii*-deficient, wild-type, and *Flii*-overexpressing mice, serum samples collected at day 50 were assessed by immunofluorescence and immunoblot analysis. Primary keratinocytes from wild-type mice were fixed and probed with pooled sera from experimental mice and staining patterns analyzed using fluorescence microscopy. Cytoplasmic and perinuclear staining within keratinocytes was apparent after incubation with sera from all three genotypes, however, only sera from OVA-induced AD-like disease *Flii^Tg/Tg^* mice produced strong nuclear staining patterns (Figure 6A). To further elucidate autoreactivity in these samples, keratinocyte whole-cell lysates were separated *via* SDS-PAGE, transferred to nitrocellulose membrane, and probed with pooled sera (Figure 6B). Additionally, autoreactivity patterns of individual mouse sera are shown in Figure S1 in Supplementary Material. Regions of differential autoreactivity were detected at 145, 70, 60, and 43 kDa band sizes (black arrows). Immunoblot analysis of sera (Figure 6C) from individual mice found that autoreactivity was highest in wild-type mice (50%) and *Flii^Tg/Tg^* mice (43%), while Flii-deficient (*Flii^+/−^)* mice showed the lowest degree of autoreactivity (25%). Positive autoreactive bands at 70 kDa were also different in *Flii^Tg/Tg^* mice compared to the two other genotypes, which may be responsible for the nuclear staining pattern observed in immunofluorescence analysis. Immunoblot analysis of sera from normal non-dermatitis mice from each genotype were also analyzed; no autoreactivity was detected (Figure S2 in Supplementary Material).

## Discussion

Flii is a cytoskeletal protein with important roles in skin development, tight junction function, skin barrier establishment, and recovery post injury which is upregulated in response to tissue inflammation and wounding (22, 31). The secreted form of Flii has been shown to affect innate immune signaling pathways and modulate cell activity and cytokine secretion from fibroblasts and macrophages *in vitro* (17, 20). Recent studies have described the contribution of *Flii* over-expression in exacerbation of inflammatory conditions, including psoriasiform dermatitis (24) and an autoimmune inflammation-mediated epidermolysis bullosa acquisita (23, 32).

To determine if Flii affects AD development and severity, mice with low (*Flii^+/−^*), normal (*Flii^+/+^*), and high (*Flii^Tg/Tg^*) expression of the *Flii* gene were exposed to repeated epicutaneous exposure to OVA and induced to form AD-like skin lesions. In contrast to our original hypothesis, *Flii* over-expression did not result in a more exacerbated development of erythema and AD-like skin lesions. However, reduced Flii levels in *Flii*-deficient mice led to significantly decreased development of AD-like skin lesions as marked by decreased erythema, TEWL, epidermal hyperplasia, and lymphocytic and mast cell tissue infiltrate compared to both wild-type and *Flii^Tg/Tg^* mice. Despite increased IFN-γ levels, the AD lesions of *Flii^+/−^* mice had reduced IL-4, which may contribute significantly to the reduced skin thickening in this model of AD (33). These findings are in agreement with studies which demonstrated that reducing Flii expression, either genetically or using Flii neutralizing antibodies, decreased tissue inflammation, and disease severity in mouse models of psoriasiform dermatitis and epidermolysis bullosa acquisita (23, 24, 32).

Genetically modified mice engineered to over-express Th_2_ cytokines develop skin barrier defects and AD spontaneously (34). The formation of AD lesions is known to be triggered by production of Th_2_ cytokines by mast cells and CD4^+^ T cells which also promote IgE production by B cells, while Th1 cells secrete IFN-γ to suppress proliferation of Th_2_ cells and IgE synthesis (35, 36). The dominance of Th_2_ cytokines in AD cause decreased expression of fillagrin and other barrier promoting molecules found in the skin (3). Examining the levels of cytokines and chemokines produced in AD-like lesional skin of *Flii^+/−^*, wild-type, and *Flii^Tg/Tg^* mice revealed decreased IFN-γ levels in *Flii^Tg/Tg^* mice suggestive of Th_2_ immune responses, while *Flii^+/−^* had increased IFN-γ levels and decreased clinical AD severity suggestive of potential Flii effect on IgE synthesis, however, this is yet to be investigated. Analysis of T-helper subsets within lesional skin showed a significantly Th_2_ skewed response in OVA-induced AD-like lesions of *Flii^Tg/Tg^* mice with higher numbers of CD4^+^GATA3^+^ cells compared to AD-like lesions of *Flii^+/−^* or wild-type counterparts who showed significantly higher Th_1_:Th_2_ ratio. Additionally, AD-like lesions of *Flii^Tg/Tg^* mice showed increased pro-inflammatory TNF-α cytokine levels and while TNF-α has been demonstrated to promote the AD development (37) we did not observe increased AD severity in these mice in this model of OVA-induced AD. As mast cell numbers were not significantly increased in *Flii^Tg/Tg^* mice, and T-helper cells were heavily skewed toward a Th2 phenotype within *Flii^Tg/Tg^* lesions, excess TNF-α was likely produced by other cell types such as macrophages, which is further supported by increased CCL22 chemokine levels observed in *Flii^Tg/Tg^* mice. Interestingly, previous studies have shown that secreted Flii reduces macrophage secretion of TNF-α *in vitro* (20) while *in vivo* studies using a mouse model of psoriasiform dermatitis showed reduced TNF-α levels in response to reduced Flii (24) which was not observed in this model of OVA-induced AD. These findings may reflect inherent differences between *in vivo* and *in vitro* studies, for example differences in complex *in vivo*, multicellular environments, as well as differences in different models of inflammatory skin diseases.

In agreement with previous studies demonstrating decreased tissue inflammation and inflammatory cytokine secretion in *Flii*-deficient mice (24, 32), OVA-induced AD-like skin lesions of *Flii^+/−^* mice showed a reduced inflammatory response marked by significantly increased anti-inflammatory IL-10 secretion as well as significantly increased IFN-γ and significantly reduced IL-4 levels. Indeed, IL-4 has been shown to be essential for eosinophil recruitment, Th_2_ cell differentiation, and IgE production (38). The increased CCL17 expression observed in AD lesions of *Flii^+/−^* mice compared to *Flii^Tg/Tg^* mice may be a potential mechanism to restore Th_1_/Th_2_ balance, as CCL17 has previously been shown to induce a Th_2_-dominated inflammatory reaction (39). Reduction of *Flii* levels did not alter IL-23 and IL-17A cytokine levels as previously observed in psoriasiform dermatitis (24) suggesting that Th_17_ responses are not involved in this model of AD. Despite significant differences in IFN-γ levels between three genotypes, CXCL9 chemokine levels were not altered between genotypes in this model of AD. IL-13 levels were also not significantly different between three genotypes, however, there was a trend to higher levels in *Flii^Tg/Tg^* mice OVA-induced AD-like skin lesions and IL-13 cytokine has previously been linked to autoantibody production in early rheumatoid arthritis (40).

Autoimmunity has been increasingly recognized to play part in exacerbating the severity of AD (6, 9, 12) as a consequence of both humoral and cellular and immunity (6). It is postulated that IFN-γ signaling during AD pathogenesis may promote the development of autoimmunity as IFN-γ overexpressing mice spontaneously develop autoantibodies (41, 42) and deletion of the IFN-γ receptor inhibits autoantibody production in lupus-prone mice (43). On the basis of our findings showing altered AD severity and altered Th_1_/Th_2_ responses including significantly altered IFN-γ expression with different *Flii* genotypes, we examined the effect of Flii levels on autoimmunity in OVA-induced AD-like skin lesions. The decreased severity of AD observed in *Flii^+/−^* mice also correlated with the reduced degree of autoreactivity (50% reduction vs WT), and further studies are required to investigate whether the autoantibodies from OVA induced AD-like skin mice contribute to or are a product of the observed AD-like symptoms. Immunoblot analysis of sera from normal non-dermatitis mice showed no autoreactivity suggesting that autoantibody development was disease-specific. Interestingly, both *Flii^+/−^* and wild-type mice showed similar autoantibody staining patterns and regions of differential autoreactivity. *Flii^Tg/Tg^* mice showed similar levels of autoreactivity compared to wild-type mice, however, in addition to the cytoplasmic and perinuclear staining pattern observed in all genotypes, the sera of these mice had a strong nuclear staining pattern. This pattern may indicate the presence of disease-mediating antinuclear antibodies which have been clinically associated with AD and other inflammatory skin disorders (44–46). Flii has previously been shown to affect TLR signaling pathways, both intracellularly and extracellularly, hence modulating innate inflammatory responses and directly impacting immune signaling, however, the potential role of Flii in autoimmunity has not been explored to date. Further studies are required to determine if autoreactivity in *Flii* genetic mice is a direct result of the impact of *Flii* on immune signaling or if it is secondary to increased pathology observed in these animals. In addition, determining the subtype of autoreactive immunoglobulins developed in the OVA-challenged murine model of AD would allow more comparison to be drawn to findings of previous clinical cohorts (10, 47).

While major differences between human AD and murine models have been demonstrated (47, 48), models of AD promote our understanding of the complex pathogenesis of human AD, and identify potential novel targets for design of targeted biologics (49). Here, we have demonstrated that Flii is a novel target in AD and that reducing its levels decreased the severity of AD in the ovalbumin-challenged murine model of AD. Additionally, we have examined the role of autoimmunity in this model of AD and while the exact mechanisms are yet to be identified, our results suggest that the effects of Flii upon Th_1_/Th_2_ balance and autoimmunity are important during AD pathogenesis.

## Ethics Statement

Mice were maintained according to the Australian Code for the Care and Use of Animals for Scientific Purposes under protocols approved by the Child Youth and Women’s Health Service Animal Ethics Committee (AEC916/06/2015).

## Author Contributions

AC and ZK conceived all the experiments with assistance from NS. ZK and NS carried out experiments and analysis with the assistance of HC and GY. ZK, NS, and AC wrote the manuscript and all authors contributed to the manuscript preparation and approved the final submitted and published versions.

## Conflict of Interest Statement

IP associated with this project has been filed by AbRegen Pty Ltd., of which AC is a shareholder and both AC and ZK are named inventors on associated patents. The handling Editor declared a past co-authorship with one of the authors AC.

## Supplementary Material

Table S1 Primer sequences used in real-time qPCR.
