# Supplementary figures and images for: Flightless I Alters the Inflammatory Response and Autoantibody Profile in an OVA-Induced Atopic Dermatitis Skin-Like Disease
Source: Front Immunol. 2018 Aug 10;9:1833. doi: 10.3389/fimmu.2018.01833 (PMC6095979; doi:10.3389/fimmu.2018.01833)

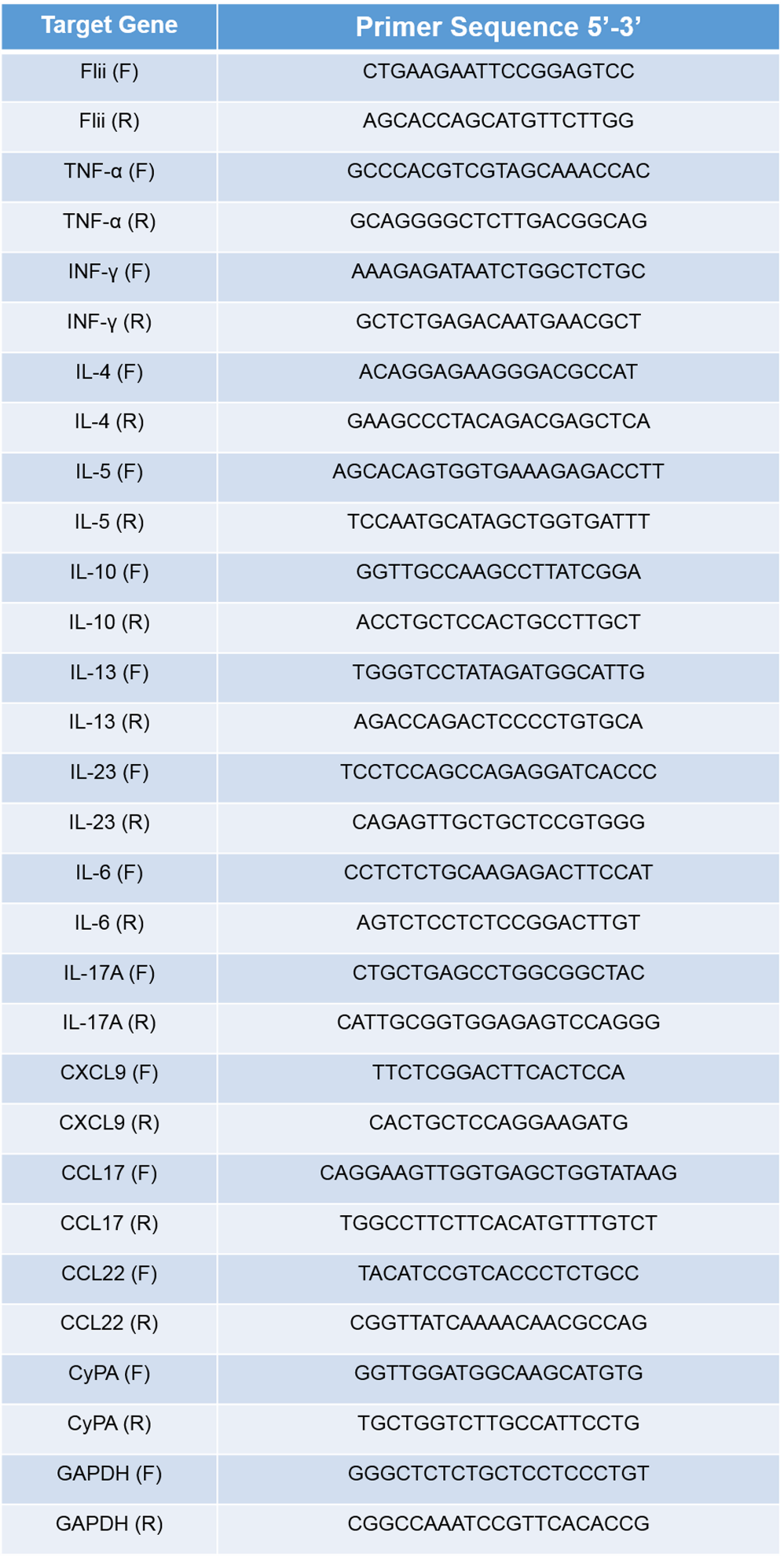

Supplement: Table S1 — Primer sequences used in real-time qPCR. [file table_1.docx]

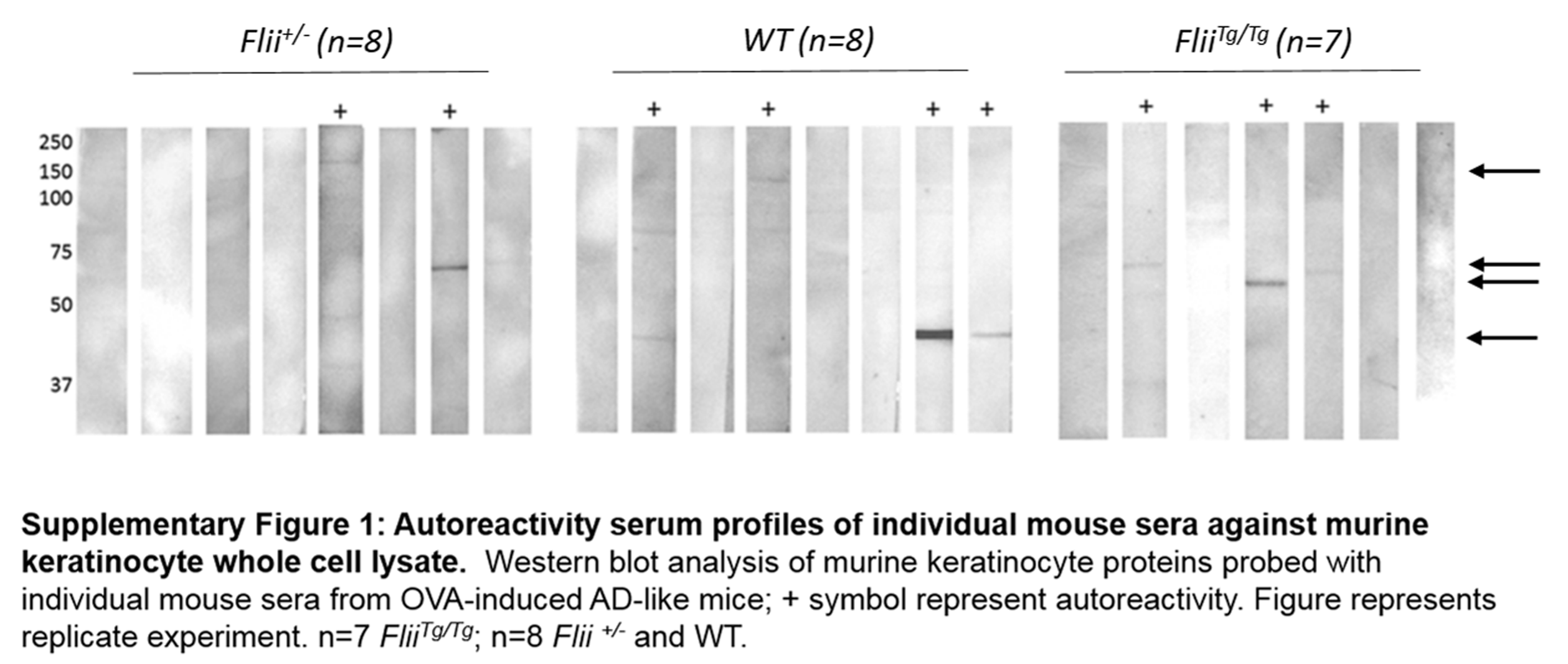

Supplement: Supplementary file 2 [file image_1.tif]

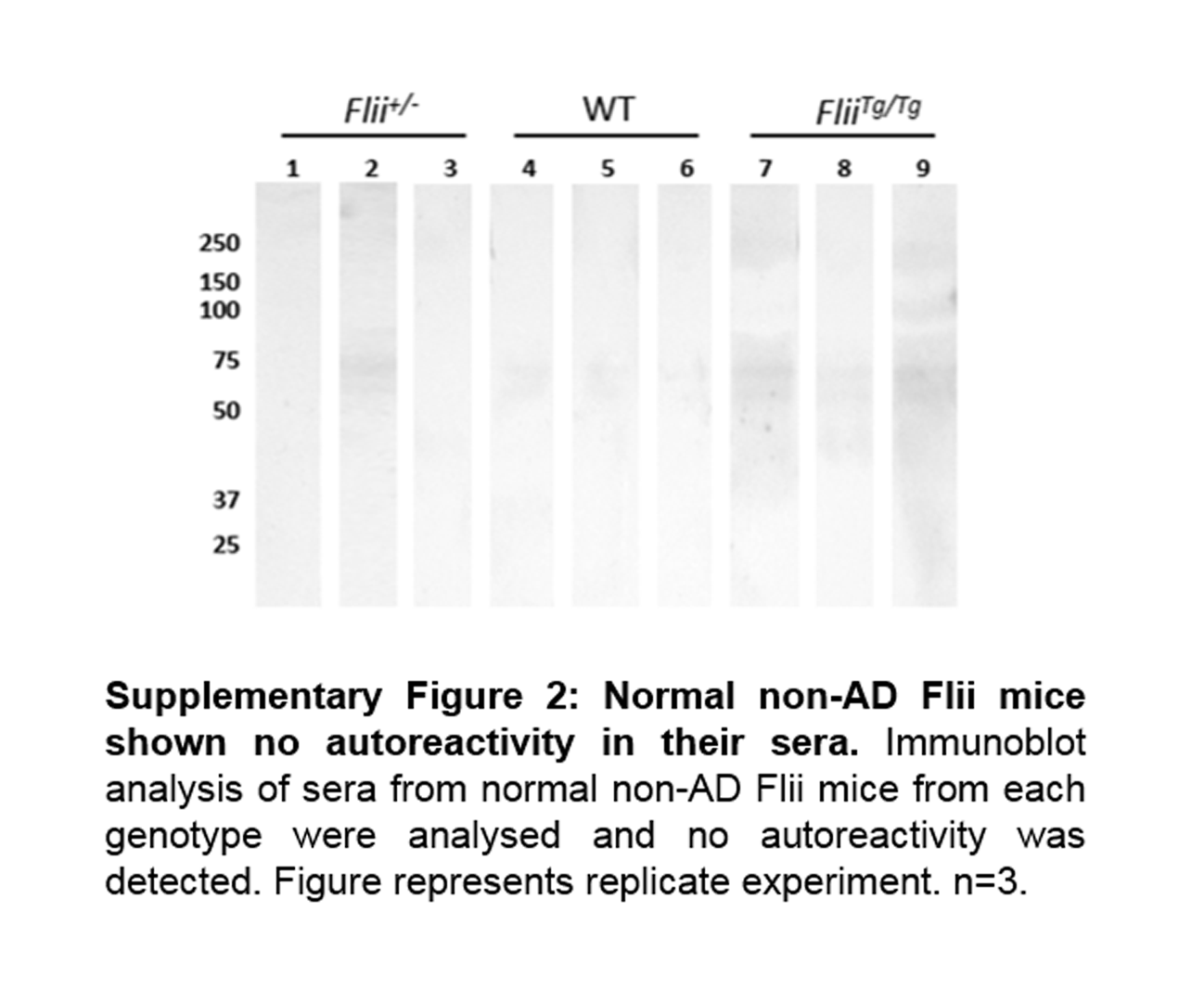

Supplement: Supplementary file 3 [file image_2.tif]
